# Resprouting trees drive understory vegetation dynamics following logging in a temperate forest

**DOI:** 10.1038/s41598-020-65367-5

**Published:** 2020-06-08

**Authors:** Radim Matula, Radomír Řepka, Jan Šebesta, Joseph L. Pettit, Juliette Chamagne, Martin Šrámek, Katherine Horgan, Petr Maděra

**Affiliations:** 1 Department of Forest Ecology, Faculty of Forestry and Wood Sciences, Czech University of Life Sciences Prague, Kamýcká 129, 165 00 Prague, Czech Republic; 2 Department of Forest Botany, Dendrology and Geobiocoenology, Faculty of Forestry and Wood Technology, Mendel University, Zemědělská 3, 613 00 Brno, Czech Republic; 3 Forest Management and Development Group, Department of Environmental System Sciences, ETH Zurich, Universitätstrasse 16, 8092 Zurich, Switzerland; 4 Institute of Evolutionary Biology and Environmental Studies, University of Zurich, Winterthurerstrasse 190, CH-8057 Zurich, Switzerland

**Keywords:** Forest ecology, Plant ecology

## Abstract

Removal of canopy trees by logging causes shifts in herbaceous diversity and increases invasibility of the forest understory. However, disturbed (cut) trees of many species do not die but resprout from remaining parts. Because sprouts develop vigorously immediately after disturbances, we hypothesized that sprouts of logged trees offset the changes in species richness and invasibility of the herbaceous layer by eliminating the rise in the resource availability during the time before regeneration from seeds develops. To test this, we analyzed data on herbaceous vegetation and sprout biomass collected in a broadleaved temperate forest in the Czech Republic before and for 6 years after logging. Sprouts that were produced by most of the stumps of logged trees offset large rises in species richness and cover of herbaceous plants and the resource availability that followed logging, but they affected the alien plants more significantly than the native plants. The sprouting canopy effectually eliminated most of the alien species that colonized the forest following a logging event. These findings indicate that in forests dominated by tree species with resprouting ability, sprouts drive the early post-disturbance dynamics of the herbaceous layer. By offsetting the post-disturbance vegetation shifts, resprouting supports forest resilience.

## Introduction

Disturbances are a major driver of forest dynamics^1,2^. In temperate forests, disturbances not only shape the dominant tree layer^3^ but, by altering understory environmental conditions, they also strongly influence the herbaceous layer^4–7^, a key source of plant diversity, as this is where the vast majority of forest plant species reside^8^.

Logging and other disturbances that remove a significant proportion of forest canopy cause a sudden increase in resource availability in the understory^9^ triggering rapid shifts in diversity and composition of the herbaceous layer^10–13^. However, many disturbed trees do not die but recover by resprouting even when their above-ground biomass is removed^14–17^. Due to the established root system and hence greater water and nutrient availability, the sprouts usually grow faster than regeneration from seeds^18,19^. Also, unlike seed regeneration, sprouts start to develop vigorously from tree remnants or roots of disturbed trees immediately following a disturbance^20^. The rapid sprout growth gives resprouting tree species a competitive advantage over slowly developing regeneration from seeds in post-disturbance forest development^18,19,21^ and allows them to form and dominate the new post-disturbance forest canopy^17,22,23^. Therefore, fast-growing sprouts may, at least partially, restore the pre-disturbance understory conditions and offset the vegetation changes caused by disturbances during the time before regeneration from seeds develops. However, despite the high frequency of resprouting trees in disturbed broadleaved forests^24^, their role in post-disturbance forest dynamics and diversity is poorly understood. In addition, resprouting is a key regeneration mode in short-rotation coppicing^25,26^, the oldest silvicultural management in the world, which has become an increasingly popular tool to support biodiversity in temperate forests^16,27–29^ but the relationship between sprout abundance and understory plant diversity remains poorly understood.

The increase in resource availability following disturbances usually increases plant species richness^4^ but also strongly increases the vulnerability of the resident plant community to invasions^30,31^, causing a steep increase in the number and abundance of alien (non-native) plant species^32,33^. Nevertheless, common forest development models^1,34,35^ state that the post-disturbance increase in plant diversity is followed by a diversity decline back to pre-disturbance or even lower diversity levels as the new canopy develops, tree density increases, and the amount of available resources declines. Because many alien plants tend to be more resource-demanding than native ones^30,31^, this decline in resource availability caused by regrowth of the canopy should affect alien plants more than native species, and it may also help to restore the high resistance of the understory to invasion typical for forests with a developed forest canopy^36–38^.

Following the initial removal of the canopy, the herbaceous layer is most affected by the speed of regeneration of emerging woody plants^39^. In forests with a high relative abundance of trees with resprouting ability, a trait common in broadleaved tree species^15^, the sprouts produced immediately after disturbances are likely to be the first woody vegetation to form a canopy. Therefore, sprouts are likely to significantly interact with herbaceous species, especially in the period prior to the development of seedlings into more advanced stages. Moreover, thanks to established root systems and resources stored in the remnant tree parts, resprouting trees grow rapidly and likely use up large amounts of available light and nutrient resources, and thus may drive the predicted decline in the understory plant diversity. In addition, by reducing resource availability, resprouters may close the temporal window of opportunity for alien colonizers^40^ typically created by disturbances^30^ and may also eliminate the alien plants that managed to establish during this window. Interestingly, despite a large body of evidence that resprouting is common as a regeneration mode of forest trees after disturbances^41–43^ and that resprouters often form a large proportion of tree regeneration^24^, no study has explored the likely significant effects of resprouting trees on the development of herbaceous plant diversity and resource availability after forest disturbances.

In this contribution, we test the hypothesis that resprouting trees drive the early development of herbaceous vegetation following logging in an oak-dominated temperate forest. We simultaneously measured sprout growth and sampled herbaceous vegetation in a coppice restoration experiment in the Czech Republic for 7 years. Specifically, we predicted that both herb species richness and cover would increase shortly after logging but then would be driven back down by growing sprout abundance as soon as sprouts developed. We also hypothesized that both logging and sprout growth would affect species richness and cover of alien plants more than those of native plants. Because we expected that changes in herbaceous vegetation would follow changes in understory environmental conditions, we predicted that logging would increase the resource availability, but growing sprouts would at least partially offset this increase. All hypotheses were tested during the period before significant seed regeneration developed.

## Results

### Temporal dynamics of the herb layer

**Figure 1:**
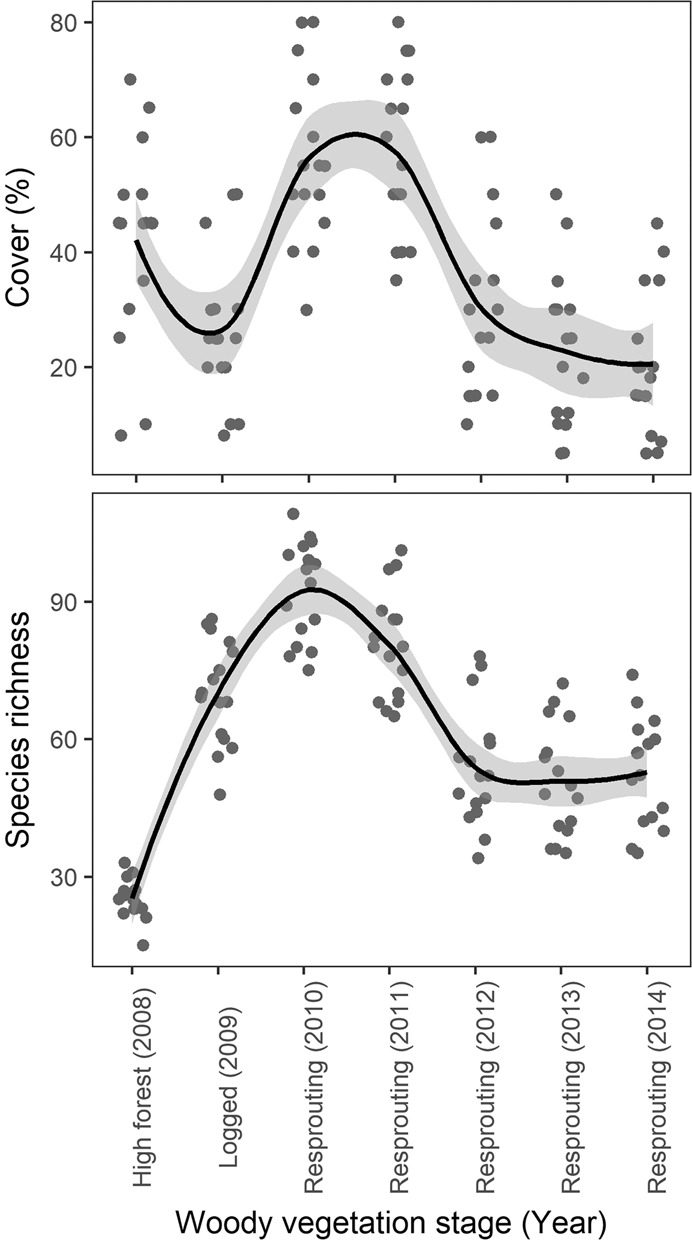
Temporal development of species richness and cover of the herbaceous layer from the vegetation season before logging (2009) to the 6th vegetation season after logging (2014). Solid line with the grey band represents the LOESS fitted model with 95% confidence intervals.

In total, we found 266 plant species, of which 215 were native and 51 alien. No alien species occurred in high forests prior to the logging event when species richness (SR) of natives was also lowest (Fig. 1). SR increased sharply following logging for two consecutive years (P < 0.001) but then declined steeply (P < 0.001) before levelling out (Fig. 1). Unlike SR, cover initially decreased (P < 0.001) but then also rose (P < 0.001) and declined again (P < 0.001) within 4 years following logging and then did not change (Fig. 1).

### Effects of changes in tree density

The decrease in tree density due to logging explained a large proportion of the initial increase in SR and cover (Table 1; SCs: SR = 0.57 ± 0.06, cover = 0.265 ± 0.07, P < 0.001) but it influenced alien plants (SCs: SR = 2.71 ± 0.38, cover = 0.93 ± 0.11) more than native plants (SCs: natives SR = 0.51 ± 0.06, cover = 0.19 ± 0.07).

**Table 1:** Proportion of the variance (R^2^) in species richness, cover, and Ellenberg indicator values (EIVs) in the forest understory explained by logging-induced changes in tree density and subsequent sprout growth.

Predictors		R^2^ - Species richness			R^2^ - Plant cover			R^2^ - EIVs		
	All	Native	Alien	All	Native	Alien	Light	Fertility	Temp.	Moisture
Tree density	77.00	0.78	0.92	0.27	0.15	0.70	0.79	0.47	0.51	0.14
Sprout growth	0.59	0.50	0.67	0.38	0.34	0.70	0.50	0.50	0.11	0.14

Variation in tree density across plots created by different logging intensities did not affect either SR or cover within any individual year (P ≥ 0.09).

### Effects of sprout growth

**Figure 2:**
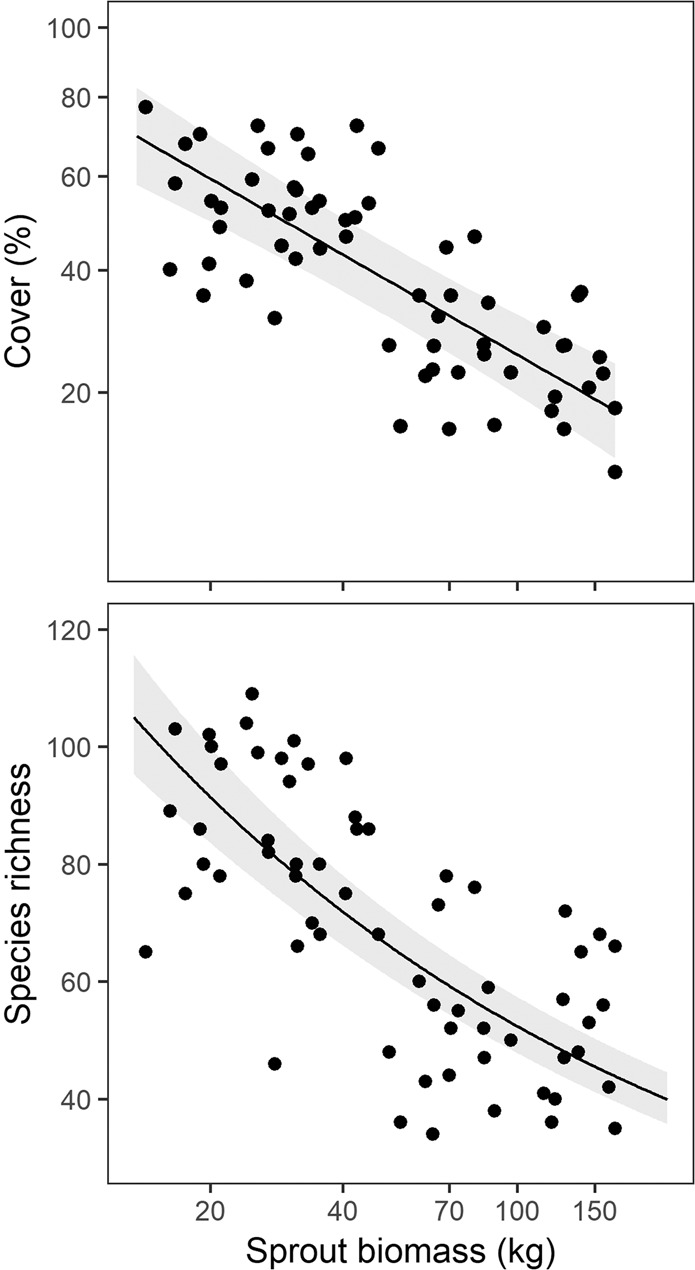
Effects of sprout biomass on species richness and cover of herbaceous vegetation in the period of significant influence (from 2nd VS to 4^th^ VS after logging). Solid lines within the grey bands represent a fitted linear mixed-effects models with 95% confidence intervals.

**Figure 3:**
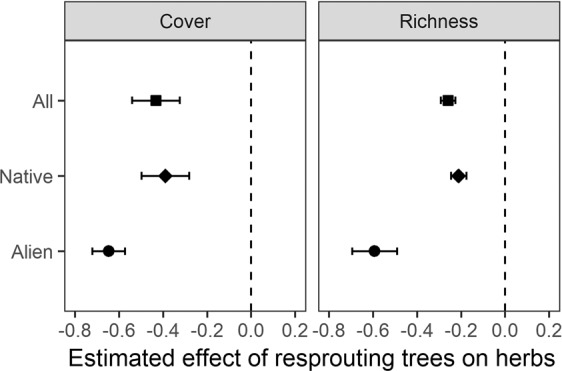
Estimated effects of resprouting trees (sprout biomass) on cover and species richness of all, alien and native plants. All variables were standardized to zero mean and unit variance. Points correspond to slope estimates and lines to 95% confidence intervals. Filled symbols indicate effects with P ≤ 0.05.

Developing sprouts decreased both SR and cover (Fig. 2) but the slope of this negative effect decreased in a log-linear fashion with increasing sprout biomass, levelling out at around 1–2 t/ha (Supplementary Fig. S2), which corresponded with the 4^th^ VS after logging. Growing sprout biomass also explained a large proportion of the observed declines in SR and cover of herbs (Table 1), but both the slopes and R^2^ values of this effect were substantially larger for alien species than for native ones (Table 1, Fig. 3).

Similar to the effect of tree density, the variation in sprout biomass across the plots did not affect the variation in either SR or cover within any individual year (P ≥ 0.11).

### Effects on environmental variables

**Figure 4:**
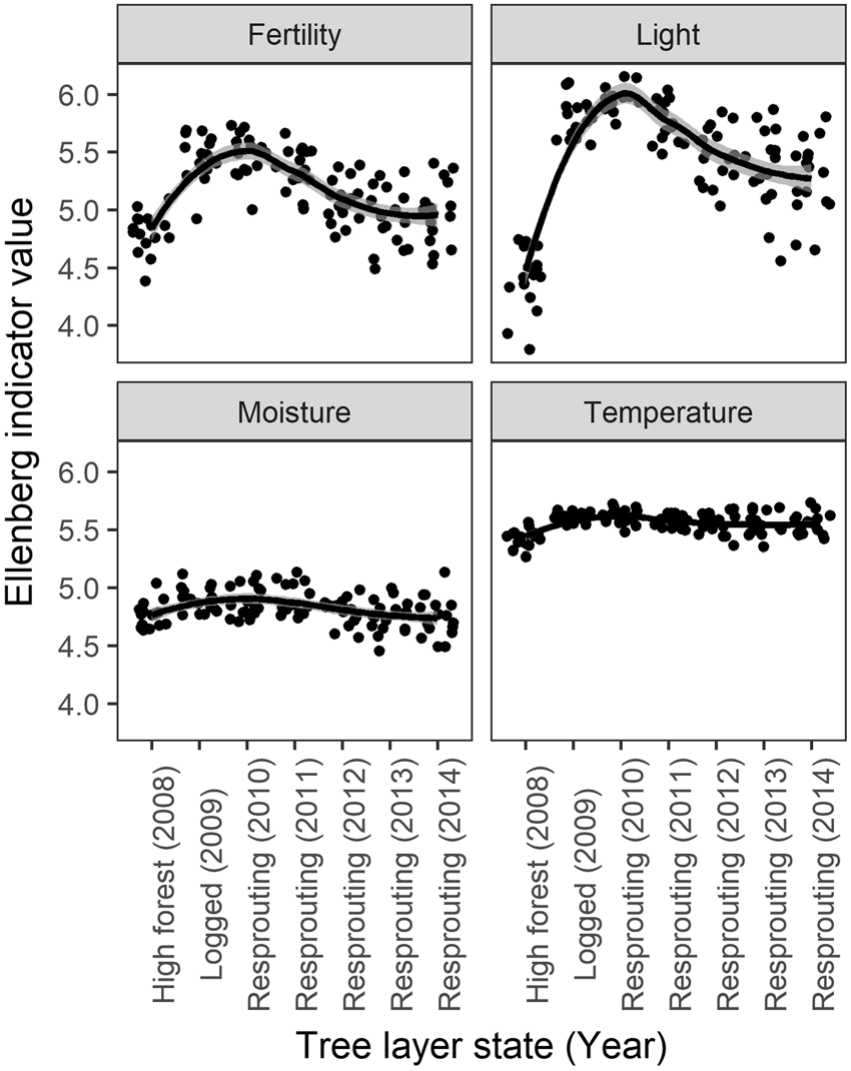
Temporal dynamics of Ellenberg indicator values for environmental variables over the study period (2008–2014). Solid lines within the grey bands represent a fitted LOESS model with 95% confidence intervals.

Although all EIVs changed over the study period, EIVs for light and soil fertility had more pronounced dynamics than EIVs for moisture and temperature (Fig. 4), which resembled the temporal dynamics of SR of herbs (Fig. 1). Both light and fertility increased after logging, then decreased and then did not change in the last two vegetation seasons (Fig. 4). In comparison to the pre-logged high forest (in 2008), EIVs for light, fertility and temperature at the end of the study period (2014) were higher (P < 0.01) but moisture did not differ (P = 0.720).

**Figure 5:**
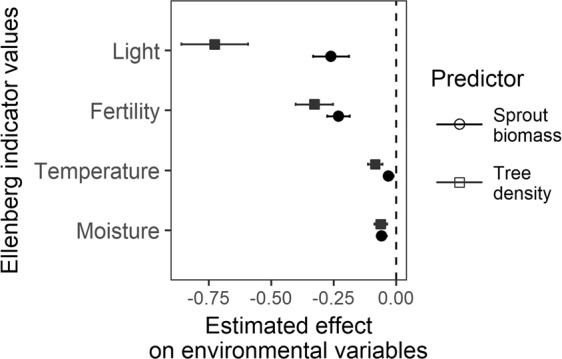
Effects of logging-induced changes in tree density and growth-induced changes in sprout biomass on environmental variables represented by Ellenberg indicator values. All variables were standardized to zero mean and unit variance. Points correspond to slope estimates and lines to 95% confidence intervals. Filled symbols indicate effects with P ≤ 0.05.

Changes in tree density and sprout biomass affected all tested EIVs, but both influenced light and fertility more significantly than other environmental variables (Fig. 5, Table 1). The effect of tree density on light was greater than the effect on fertility, whereas sprout biomass had a similar negative effect on both light and fertility (Fig. 5).

EIVs had a significant positive relationship with both SR and cover of all plant groups (Supplementary Table S2), but the effect of light was by far the largest followed by the effect of fertility, whereas the effects of temperature and moisture were significantly smaller (Supplementary Table S2\).

## Discussion

Our study demonstrated that sprouts produced from remnants of disturbed, in our case logged, trees offset the large shifts in the herbaceous vegetation and the understory resource availability caused by canopy-removing disturbances. Even though the resprouting trees reduced the initial massive rise in herbaceous diversity and cover, they, as we expected, affected alien plants more significantly than native plants, eliminating most of the alien species that colonized the previously alien-species free forest. Our novel tests on how resprouters influence early post-disturbance forest dynamics support our hypothesis that trees species with resprouting ability drive the early post-disturbance dynamics of the herbaceous layer, at least in the period before regeneration from seeds establishes and reaches competitive size. It is also evident that, by offsetting the typical post-disturbance increases in resource availability and by filtering out the non-native species within a short time after disturbances, the resprouters significantly increase forest resilience.

As we predicted, the decrease in tree density due to logging initially caused a steep rise in the number and cover of both alien and native species, which agrees with the effects of logging previously reported by several authors^11,28,44^. However, comparable studies that have also documented the subsequent species richness decline and what drives it are scarce. The pattern of diversity dynamics observed in our study agrees with Strubelt *et al*.^29^, who also found a diversity increase followed by a diversity decrease within a short period of time after logging in coppiced forests. Although they did not relate these diversity dynamics to woody layer development, sprouts dominate woody plant regeneration in coppices^45^ and the similar diversity dynamics they observed were probably also driven by sprout development. Belote *et al*.^11^ compared understory vegetation shortly after logging to 9 years after logging in temperate forests in the USA and found that the number of non-native species decreased with time after logging whereas the number of native species did not change. The steeper decline in alien plants supports our hypothesis that the newly developing woody layer affects alien herbaceous species more than native herbaceous species, probably due to the greater resource requirements of the alien species. However, Soler *et al*.^46^ found increasing species richness of alien plants in a temperate *Nothofagus pumilio* forest even 8 years after logging. Nevertheless, tree regeneration in this area is very slow, with new stems reaching only 30–40 cm in height after 8 years^47^, which may yet be too small to limit the colonization of the new invaders. In comparison, the resprouting trees in our study plots were already 7.5 m tall (on average) 7 years after logging and formed a closed canopy (Matula, unpublished data).

The observed rise and subsequent decline in species richness in our study is also in line with the forest succession models which predict: a newly developing woody layer will eliminate the initial post-disturbance rise in species richness due specifically to decreasing resource availability^35^. But, because the pace of the depletion of resources released by a disturbance is driven by the pace of tree canopy formation^48^, we assume that the timing and the speed of the decline in species richness differs between forest stands with resprouting woody species and stands without them (e.g. coniferous forests). In our sessile oak-dominated forests, most of the logged trees resprouted, and to a great extent restored diversity and resource availability within 4 years after logging. This was also the point at which regeneration from seeds had developed to the seedling stage (Matula, unpublished data). Halpern^12^ observed that the recovery of forest understories from severe disturbances took significantly more time in forests that regenerated purely from seed than in forests where at least some species resprouted. This pattern implies that a phase of decline in species richness and resource availability occurs significantly sooner in forests with resprouters than in forests without them. Through rapid resource depletion, the rapidly growing resprouting trees in our study closed the post-disturbance window of opportunity for alien colonizers within a few years after logging. This suggests that resprouters make the window of opportunity significantly shorter than regeneration from seed, therefore making forests less vulnerable to disturbance triggered invasions.

The positive correlation between the number of harvested trees and sprout biomass in our study suggests that the more trees (with sprouting ability) that are removed, the greater the abundance of sprouts and in turn the faster the restoration of understory conditions occurs, indicating that resprouters may offset the effect of higher harvesting intensity on understory plant diversity^49^. However, we speculate that this relationship is probably valid only until a certain logging intensity threshold because if logging is done in a very large area and with greater intensity, resprouters may not be able to offset understory vegetation changes quickly enough and to as great an extent. The relatively large influence of resprouters on the understory, despite a significant proportion (24.6%) of the harvested trees failing to resprout, suggests that sprouts may also affect understory development in forest stands composed of a mix of resprouters and reseeders, such as in common mixtures of resprouting broadleaved species and non-sprouting coniferous species^12^. It should be noted, however, that there is a decline in resprouting success of trees with increasing neighborhood densities^16^. Trees failing to resprout are more likely to be near trees that successfully resprouted. Thus, even if a proportion of the disturbed trees fail to resprout, the influence of these trees is likely buffered by neighbors that will fill unused spaces left by non-sprouting stems.

Our results also demonstrate that sprouts affected herbaceous plants only in their early development. Sprouts showed a significant influence on cover and diversity up until they reached 1–2t biomass per ha (i.e. until 4 vegetation seasons after the logging event), but greater sprout biomasses than this had no evident effects (Supplementary Fig. S2). Similarly, resources (especially light and nutrients) declined steeply only until the 4th vegetation season after logging (Fig. 4) as did the species richness and cover of both native and alien plants (Fig. 1), which supports our hypothesis that the sprout-related changes in the understory vegetation are mediated through changes in resource availability. In this experiment, we did not directly measure light or nutrient levels, the most influential environmental variables, but we detected significant growth-limiting neighborhood competition among resprouting trees in the same plot by the third vegetation season after harvesting (Matula, unpublished data). This suggests that, by this time, resprouting trees had used up most of the available resources provided by harvesting and had started competing for these resources.

The significant effects of developing sprouts on understory plant diversity are especially relevant for coppice restoration projects, which frequently aim to increase diversity of understory plants^28,50,51^. It is evident that sprouts, to a large extent, offset the initial rise in diversity caused by logging, but as our study shows, a significant proportion of the newly established species remain, increasing local plant diversity, even after the new canopy is formed. This pattern is also in line with diversity changes following logging in coppices observed by Strubelt *et al*.^29^. In addition, as our results show, rapidly developing sprouts filter out more conservationally undesirable alien species, whose relative abundance is often increased by logging^52^. However, the understory species filtering done by developing sprouts seems to be ineffective against the regeneration of certain invasive alien tree species, which have been previously observed invading forest stands following traditional coppice management^53^. It should also be noted, that the significant decline in diversity observed in our study occurred despite a small but significant proportion of stumps failing to resprout, which means that the resprouting stumps could not create a fully closed canopy. Thus, in forests dominated by species with good resprouting ability, in which resprouting failure is very rare (e.g. *Tilia cordata* and *Carpinus betulus* in our plot^45^), the canopy formed by sprouts is likely to be more closed, thus causing more significant understory diversity declines. These adverse effects of sprouts may therefore potentially hamper certain conservation goals of coppice restoration projects and some other measures would be needed to maintain higher understory diversity in the long-term.

Our study demonstrates that resprouters strongly drive the early vegetation dynamics and resource availability in the understory by which they may have long-term effects on forest plant diversity during the whole successional cycle. The ability of trees to quickly restore understory conditions after disturbance by resprouting, even if only partially, decreases the susceptibility of the forest ecosystem to invasions and likely increases forest resilience to disturbances in general. Although it is not clear what will happen to the resprouting trees in the future (i.e. whether they will dominate the forest canopy for the whole successional cycle or will be outgrown by regeneration from seed), at some point and to some extent, seed regeneration will develop and may further reduce herbaceous cover and native species richness. Therefore, in forests where trees have the ability to resprout, the species richness decline caused by the development of the tree canopy in early successional stages may not be as continuous as previously shown^1,54^ but rather consists of three consequent phases: a steep but short-term diversity decline caused by resprouting trees, followed by a stable stage until advanced seed regeneration starts emerging, which when developed, triggers another diversity decline. However, a much longer-term study would be needed to confirm such a theory.

In this study, we demonstrated the significant but previously unknown ability of resprouting trees to offset the significant shifts in plant diversity and resource availability caused by logging. This indicates the important role of resprouting for forest diversity and dynamics. It also suggests that sprout development is a key driver of plant diversity in coppiced forests, where resprouting is a common regeneration mode of woody species. Our results also highlight the fact that resprouting is generally understudied in comparison to reseeding^55,56^ and further studies are needed to not only understand resprouting itself but also to understand its role in the functioning of the many ecosystems dominated by woody species where resprouting is common.

## Methods

### Site characteristics

This study was conducted in the Hády experimental plot, located in the Křtiny Training Forest Enterprise of Mendel University in Brno, south-eastern Czech Republic (49°13′30′′N, 16°40′55′′E). This is a square plot that has an area of 4 ha (200 m per 200 m). The elevation of the plot is 401 m a.s.l. The bedrock is limestone, and the soils are brown forest soils. The average annual rainfall is 510 mm, and the average annual air temperature is 8.4 °C. Average temperature in July (the warmest month) is 18.4 °C, and in January (the coldest month) is −2.1 °C, based on data from 1960–2010 from the nearest weather station, Brno. The plot is fenced because there is significant browsing pressure in the area.

The long-term objective of the Hády experimental plot is to restore traditional coppice and coppice with standard management, which was applied in the study forest in the past (until 1902)^50^. Since the high forest was planted in 1902, this forest has only been subject to one major treatment which consisted of commercial logging of the high forest in 2008/9, and no other intervention or treatment after that^45^. The original forest (before logging in 2008/2009) was approximately 103 years old with an average total basal area (BA) of 33.2 m^2^ ha^−1^ and a density of 689 trees/ha. We did not observe any legacies of past coppicing such as inflated bases or multi-stem polycormon structures^57^ on the trees in the plot. In total, twelve woody species occurred in the stand, and it was dominated by sessile oak (*Quercus petraea* (Matt.) Liebl.), small-leaved lime (*Tilia cordata* Mill.) and European hornbeam (*Carpinus betulus* L.). All the dominant tree species have good resprouting ability^20^; therefore, we expected them to produce sprouts vigorously after harvesting.

### Experimental design and woody plants measurements

In 2008, prior to logging, the area of the experimental plot was divided into 16 regular subsquares (50 by 50 m), with a permanent subplot (20 by 20 m) for sampling herbaceous vegetation (vegetation plots) in the centre of each subsquare (Supplementary Fig. S1). In each vegetation plot and in a belt 5 m around it (tree plot), diameter at breast height (DBH) and spatial position were measured for all trees with DBH ≥ 7 cm, using the Field-Map technology (IFER, Ltd., Jílové u Prahy, Czech Republic; www.fieldmap.cz). Tree plots include a 5 m buffer around vegetation plots because the influence of the canopy on understory vegetation extends beyond the boundary of the vegetation plot alone. We measured trees and later sprouts in tree plots. In the following winter (2008/2009), most of the trees in the plot were cut approximately 5–10 cm above ground level and removed from the plot. To vary the density of residual trees, 1 to 20 trees per tree plot were left uncut (averaging 21.1 m in height and 41.5 cm in DBH). Details on stand structural characteristics before and after logging are shown in Supplementary Table S1. Then, for the next 6 years, before the start of each vegetation season (from 2009 to 2014), all the cut trees were revisited, and the basal diameter of the five thickest sprouts per stump was measured for those exhibiting resprouting, a metric found to be a good proxy of total sprout biomass^58^. We checked for both stump and root sprouts, though root sprouts were not present in any tree plot. Of all harvested trees, 75.4% survived and had live sprouts in the last vegetation season. We also surveyed for stems regenerating from seed (≥1 cm in DBH), but none were found during the study period.

### Herbaceous vegetation sampling

To sample the understory vegetation, the total cover and the cover of each herbaceous species were visually estimated (as a %) for each vegetation plot in the vegetation season (VS) before logging and in the 6 VSs following logging (from 2008 to 2014). Cover was estimated as the percentage of the vegetation plot covered by herbaceous plants/species. Sampling was always completed in late June/early July when the spring and summer vegetation aspects intersected, and herb diversity peaked^59^. The vegetation surveys included all herbs and all woody plants with a height ≤1 m and were always done by the same two trained botanists.

### Environmental variables

To test the effects of logging and sprout biomass on resource availability and how this resource availability influenced herbaceous vegetation, we calculated Ellenberg indicator values (EIVs) for light, soil fertility, temperature, and moisture^60^ for each vegetation plot in each VS using cover values of the herbaceous species (weighted mean). These EIVs were then used as proxies for the environmental variables in the analyses. Using EIVs instead of measured variables is convenient because EIVs represent general environmental condition over the whole plot whereas one-time and spatially limited measured variables provide only a “snapshot” of the conditions. On the other hand, EIVs may potentially overestimate the significance of the relationship between SR and the environmental variables^61^. However, this potential overestimation was likely small in our study because it arises when samples from different species pools are analyzed together^61^, but our study was limited to one site with a common species pool.

### Quantification of abundances of woody plants

To quantify abundances of resprouting trees, we calculated above-ground woody biomass of sprouts individually for every tree in each VS, using allometric equations developed for resprouting woody plants in the same area^58^. We then summed the individual biomasses per tree plot, both for all species together and separately. Sprout biomass was correlated with the number of harvested trees in all years (Pearson r range = 0.61–0.80, P < 0.001). To quantify the effect of tree harvesting and residual (uncut) trees abundance, we calculated the number of standing trees per plot (tree density) for each tree plot for each VS.

### Data analyses

Recorded plant species were classified into natives and aliens according to Pyšek *et al*.^62^. For all species pooled together and for each of the plant groups (natives and aliens), we calculated species richness (SR) and cover for each vegetation plot in each VS. To obtain cover for the plant groups, we first calculated the proportion of each group as a ratio between the sum of covers of the species in the group and the sum of covers of all species and then multiplied the total cover (estimated independently) by these proportions.

To analyze temporal patterns in SR and cover over the whole sampling period, we used locally weighted polynomial regression (LOESS)^63^. We divided the analyses into two periods, before (pre-sprout period) and after sprouts developed (sprout period) according to the expected drivers. Before the sprouts started to grow the herbaceous vegetation could be influenced only by logging but in the later period, when the sprout appeared, and the tree density remained unchanged, the rapidly increasing sprout abundance (biomass) was likely to be the main driver of changes in the herbaceous layer.

All the hypothesized effects were tested using models with either SR, cover or EIV as a response variable and either tree density as an explanatory variable in models for the pre-sprout period or sprout biomass as explanatory variables in models for the sprout period. Tree density was not used in models for the sprout period because it did not change during this period (mean: ∆tree density ≤ 0.5%). For models with SR as a response variable, we used generalized linear mixed models with Poisson error distribution and plot identity as a random effect. To test effects on covers and EIVs we employed general linear models also with a random effect of plot identity. These mixed models with the random effect of plot identity were employed to test mainly the temporal effects of changes in tree density (due to logging) and sprout growth. To quantify the effect of varying tree density and sprout biomass (created by different intensities of logging) on SR and cover of herbaceous plants within individual years, we used models with the same response and explanatory variables as in the mixed models described above but with only data for given year and without random effects (i.e. general and generalized linear models). Because models with BA had a consistently worse fit than models with tree density (∆AIC ≥ 4.1), we present only results for the effects of tree density. All explanatory variables were standardized to zero mean and unit variance. Cover and sprout biomass were log-transformed to normalize residuals. For each model, we calculated R^2^s. For mixed models, we computed conditional R^2^ using the method of Nakagawa and Schielzeth^64^ with the extension of Johnson^65^ and Nagelkerke’s R^2^^66^ for generalized linear models, both calculated with the “MuMIn” package version 1.42.1. All the analyses were carried out in R version 3.5.1^67^. For all mixed models, we used the “lme4” package version 1.1–18–7.

## Supplementary information

Supplementary information.

Supplementary Table S1.

## Data Availability

All data generated and analyzed during this study are included in this published article (and its Supplementary Information files).

## References

[1] Franklin JF (2002). Disturbances and structural development of natural forest ecosystems with silvicultural implications, using Douglas-fir forests as an example. For. Ecol. Manage..

[2] Seidl R, Schelhaas M-J, Lexer MJ (2011). Unraveling the drivers of intensifying forest disturbance regimes in Europe. Glob. Chang. Biol..

[3] Frelich LE, Reich PB (1999). Neighborhood Effects, Disturbance Severity, and Community Stability in Forests. Ecosystems.

[4] Roberts MR (2004). Response of the herbaceous layer to natural disturbance in North American forests. Can. J. Bot..

[5] Halpern CB, McKenzie D, Evans SA, Maguire DA (2005). Initial responses of forest understories to varying levels and patterns of green-tree retention. Ecol. Appl..

[6] Royo AA, Collins R, Adams MB, Kirschbaum C, Carson WP (2010). Pervasive interactions between ungulate browsers and disturbance regimes promote temperate forest herbaceous diversity. Ecology.

[7] Duguid MC, Frey BR, Ellum DS, Kelty M, Ashton MS (2013). The influence of ground disturbance and gap position on understory plant diversity in upland forests of southern New England. For. Ecol. Manage..

[8] Gilliam FS (2007). The Ecological Significance of the Herbaceous Layer in Temperate Forest Ecosystems The Ecological Significance of the Herbaceous Layer in Temperate Forest Ecosystems. Bioscience.

[9] Gutschick VP, BassiriRad H (2003). Extreme events as shaping physiology, ecology, and evolution of plants: toward a unified definition and evaluation of their consequences. New Phytol..

[10] Thomas SC, Halpern CB, Falk DA, Liguori DA, Austin KA (1999). Plant diversity in managed forests: understory responses to thinning and fertilization. Ecol. Appl..

[11] Belote RT, Jones RH, Wieboldt TF (2012). Compositional stability and diversity of vascular plant communities following logging disturbance in Appalachian forests. Ecol. Appl..

[12] Halpern CB (1988). Early successional pathways and the resistance and resilience of forest communities. Ecology.

[13] Šebesta, J., Maděra, P., Řepka, R. & Matula, R. Comparison of vascular plant diversity and species composition of coppice and high beech forest in the Banat region, Romania. Folia Geobot. 1–11 (2017).

[14] Atwood CJ, Fox TR, Loftis DL (2009). Effects of alternative silviculture on stump sprouting in the southern Appalachians. For. Ecol. Manage..

[15] Del Tredici P (2001). Sprouting in temperate trees: A morphological and ecological review. Bot. Rev..

[16] Svátek M, Matula R (2015). Fine-scale spatial patterns in oak sprouting and mortality in a newly restored coppice. For. Ecol. Manage..

[17] Dietze MC, Clark JS (2008). Changing the gap dynamics paradigm: vegetative regeneration control on forest response to disturbance. Ecol. Monogr..

[18] Larsen DR, Johnson PS (1998). Linking the ecology of natural oak regeneration to silviculture. For. Ecol. Manage..

[19] Swaim TJ (2016). Predicting the height growth of oak species (Quercus) reproduction over a 23-year period following clearcutting. For. Ecol. Manage..

[20] Matula R (2012). The sprouting ability of the main tree species in Central European coppices: implications for coppice restoration. Eur. J. For. Res..

[21] Clarke PJ (2013). Resprouting as a key functional trait: how buds, protection and resources drive persistence after fire. New Phytol..

[22] Brudvig LA, Asbjornsen H (2007). Stand structure, composition, and regeneration dynamics following removal of encroaching woody vegetation from Midwestern oak savannas. For. Ecol. Manage..

[23] Shure DJ, Phillips DL, Edward Bostick P (2006). Gap size and succession in cutover southern Appalachian forests: an 18 year study of vegetation dynamics. Plant Ecol..

[24] Elliott KJ, Knoepp JD (2005). The effects of three regeneration harvest methods on plant diversity and soil characteristics in the southern Appalachians. For. Ecol. Manage..

[25] Volařík, D. *et al*. Variation in canopy openness among main structural types of woody vegetation in a traditionally managed landscape. Folia Geobot. 1–18 (2017).

[26] Dinh TT (2019). Stump sprout dynamics of Quercus serrata Thunb. and Q. acutissima Carruth. four years after cutting in an abandoned coppice forest in western Japan. For. Ecol. Manage..

[27] Kadavý J, Kneifl M, Knott R (2011). Establishment and selected characteristics of the Hády coppice and coppice-with-standards research plot (TARMAG I). J. For. Sci..

[28] Vild O, Roleček J, Hédl R, Kopecký M, Utinek D (2013). Experimental restoration of coppice-with-standards: Response of understorey vegetation from the conservation perspective. For. Ecol. Manage..

[29] Strubelt I, Diekmann M, Griese D, Zacharias D (2019). Inter-annual variation in species composition and richness after coppicing in a restored coppice-with-standards forest. For. Ecol. Manage..

[30] Davis MA, Grime JP, Thompson K (2000). Fluctuating resources in plant communities: a general theory of invasibility. J. Ecol..

[31] Blumenthal D (2005). Ecology. Interrelated causes of plant invasion. Science.

[32] Davis MA, Thompson K, Grime JP (2005). Invasibility: The local mechanism driving community assembly and species diversity. Ecography (Cop.)..

[33] Funk JL, Vitousek PM (2007). Resource-use efficiency and plant invasion in low-resource systems. Nature.

[34] Oliver CD (1980). Forest development in North America following major disturbances. For. Ecol. Manage..

[35] Roberts, M. R. & Gilliam, F. S. Disturbance effects on herbaceous layer vegetation and soil nutrients in Populus forests of northern lower Michigan. J. Veg. Sci. 903–912 (1995).

[36] Rejmánek, M. Invasibility of plant communities. In *Biological invasions: a global perspective* 369–388 (1989).

[37] Von Holle B, Delcourt HR, Simberloff D, Harcombe P (2003). The importance of biological inertia in plant community resistance to invasion. J. Veg. Sci..

[38] Whitfeld TJS, Lodge AG, Roth AM, Reich PB (2014). Community phylogenetic diversity and abiotic site characteristics influence abundance of the invasive plant Rhamnus cathartica L. J. Plant Ecol..

[39] Davis MA, Wrage KJ, Reich PB (1998). Competition between tree seedlings and herbaceous vegetation: support for a theory of resource supply and demand. J. Ecol..

[40] Myster RW (1993). Tree invasion and establishment in old fields at Hutcheson Memorial Forest. Bot. Rev..

[41] Pyttel PL, Fischer UF, Suchomel C, Gärtner SM, Bauhus J (2013). The effect of harvesting on stump mortality and re-sprouting in aged oak coppice forests. For. Ecol. Manage..

[42] Keyser T, Loftis D (2015). Stump sprouting of 19 upland hardwood species 1 year following initiation of a shelterwood with reserves silvicultural system in the southern Appalachian Mountains, USA. New For..

[43] Nzunda EF, Griffiths ME, Lawes MJ (2008). Sprouting by remobilization of above‐ground resources ensures persistence after disturbance of coastal dune forest trees. Funct. Ecol..

[44] Jauni M, Gripenberg S, Ramula S (2015). Non‐native plant species benefit from disturbance: a meta‐analysis. Oikos.

[45] Matula R (2019). Pre-disturbance tree size, sprouting vigour and competition drive the survival and growth of resprouting trees. For. Ecol. Manage..

[46] Soler RM, Schindler S, Lencinas MV, Peri PL, Pastur GM (2016). Why biodiversity increases after variable retention harvesting: A meta-analysis for southern Patagonian forests. For. Ecol. Manage..

[47] Pastur GJM (2014). Survival and growth of Nothofagus pumilio seedlings under several microenvironments after variable retention harvesting in southern Patagonian forests. Ann. For. Sci..

[48] Ogle K, Pacala SW (2009). A modeling framework for inferring tree growth and allocation from physiological, morphological and allometric traits. Tree Physiol..

[49] Belote RT, Jones RH, Hood SM, Wender BW (2008). Diversity-invasibility across an experimental disturbance gradient in Appalachian forests. Ecology.

[50] Kadavý, J., Kneifl, M. & Knott, R. Biodiversity and Target Management of Endangered and Protected Species in Coppices and Coppices-with-Standards Included in System of NATURA 2000. (Mendel University in Brno, 2011).

[51] Kirby KJ (1990). Changes in the ground flora of a broadleaved wood within a clear fell, group fells and a coppiced block. Forestry.

[52] Roberts, M. R. & Gilliam, F. S. Response of the Herbaceous Layer to Disturbance in Eastern Forests. In *The Herbaceous Layer in Forests of Eastern North America* 320–339 (Oxford University Press, 2014), 10.1093/acprof:osobl/9780199837656.003.0013.

[53] Radtke A (2013). Traditional coppice forest management drives the invasion of Ailanthus altissima and Robinia pseudoacacia into deciduous forests. For. Ecol. Manage..

[54] Oliver, C. D. & Larson, B. C. Forest stand dynamics. (McGraw-Hill, Inc., 1990).

[55] Bond WJ, Midgley JJ (2001). Ecology of sprouting in woody plants: The persistence niche. Trends Ecol. Evol..

[56] Tanentzap AJ, Mountford EP, Cooke AS, Coomes DA (2012). The more stems the merrier: advantages of multi-stemmed architecture for the demography of understorey trees in a temperate broadleaf woodland. J. Ecol..

[57] Vrška T, Janík D, Pálková M, Adam D, Trochta J (2017). Below-and above-ground biomass, structure and patterns in ancient lowland coppices. IForest.

[58] Matula, R., Damborská, L., Nečasová, M., Geršl, M. & Šrámek, M. Measuring biomass and carbon stock in resprouting woody plants. PLoS One 10, (2015). 10.1371/journal.pone.0118388 PMC4342014 25719601

[59] Chamagne J (2016). Do the rich get richer? Varying effects of tree species identity and diversity on the richness of understory taxa. Ecology.

[60] Ellenberg, H. *et al*. Zeigerwerte von pflanzen in Mitteleuropa. (1992).

[61] Zelený D, Schaffers AP (2012). Too good to be true: pitfalls of using mean Ellenberg indicator values in vegetation analyses. J. Veg. Sci..

[62] Pyšek P, Sádlo J, Mandák B (2002). Catalogue of alien plants of the Czech Republic. Preslia.

[63] Cleveland WS, Devlin SJ (1988). Locally weighted regression: an approach to regression analysis by local fitting. J. Am. Stat. Assoc..

[64] Nakagawa S, Schielzeth H (2013). A general and simple method for obtaining R2 from generalized linear mixed-effects models. Methods Ecol. Evol..

[65] Johnson PCD (2014). Extension of Nakagawa & Schielzeth’s R2GLMM to random slopes models. Methods Ecol. Evol..

[66] Nagelkerke NJD (1991). A note on a general definition of the coefficient of determination. Biometrika.

[67] R Core Team. R: A language and environment for statistical computing. R Foundation for Statistical Computing. (2018).

