# Supplementary material for: Resprouting trees drive understory vegetation dynamics following logging in a temperate forest
Source: Sci Rep. 2020 Jun 8;10:9231. doi: 10.1038/s41598-020-65367-5 (PMC7280521; doi:10.1038/s41598-020-65367-5)
Supplement: Supplementary file 1 — Supplementary information. [file 41598_2020_65367_MOESM1_ESM.pdf]

Supplementary information

Resprouting trees drive understory vegetation dynamics following logging in a temperate forest

Radim Matula, Radomír Řepka, Jan Šebesta, Joseph L. Pettit, Juliette Chamagne, Martin Šrámek, Katherine Horgan, Petr Maděra

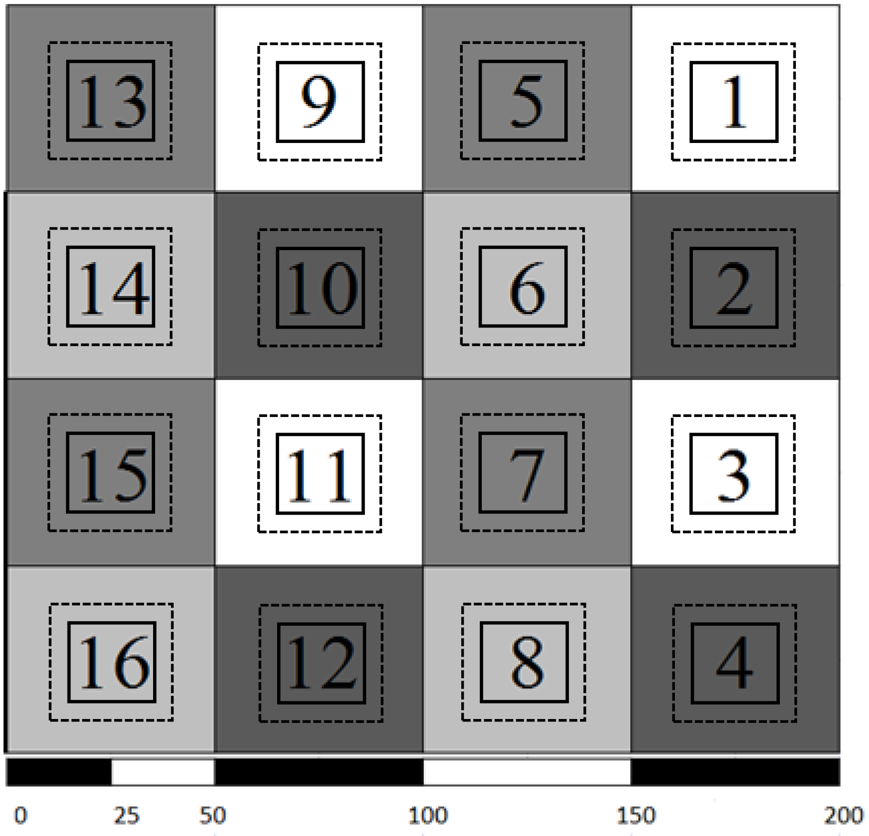

Figure S1. Design of the Hády experimental plot. The smaller sub-squares with solid-line border with numbers represent vegetation plots, where herbaceous species were sampled; the dashed square around them represent area, where trees and sprouts were measured. Different greyscale of the squares represents different density of uncut trees; the darker the more trees were left. Details on tree density in the vegetation plots are shown in table S1.

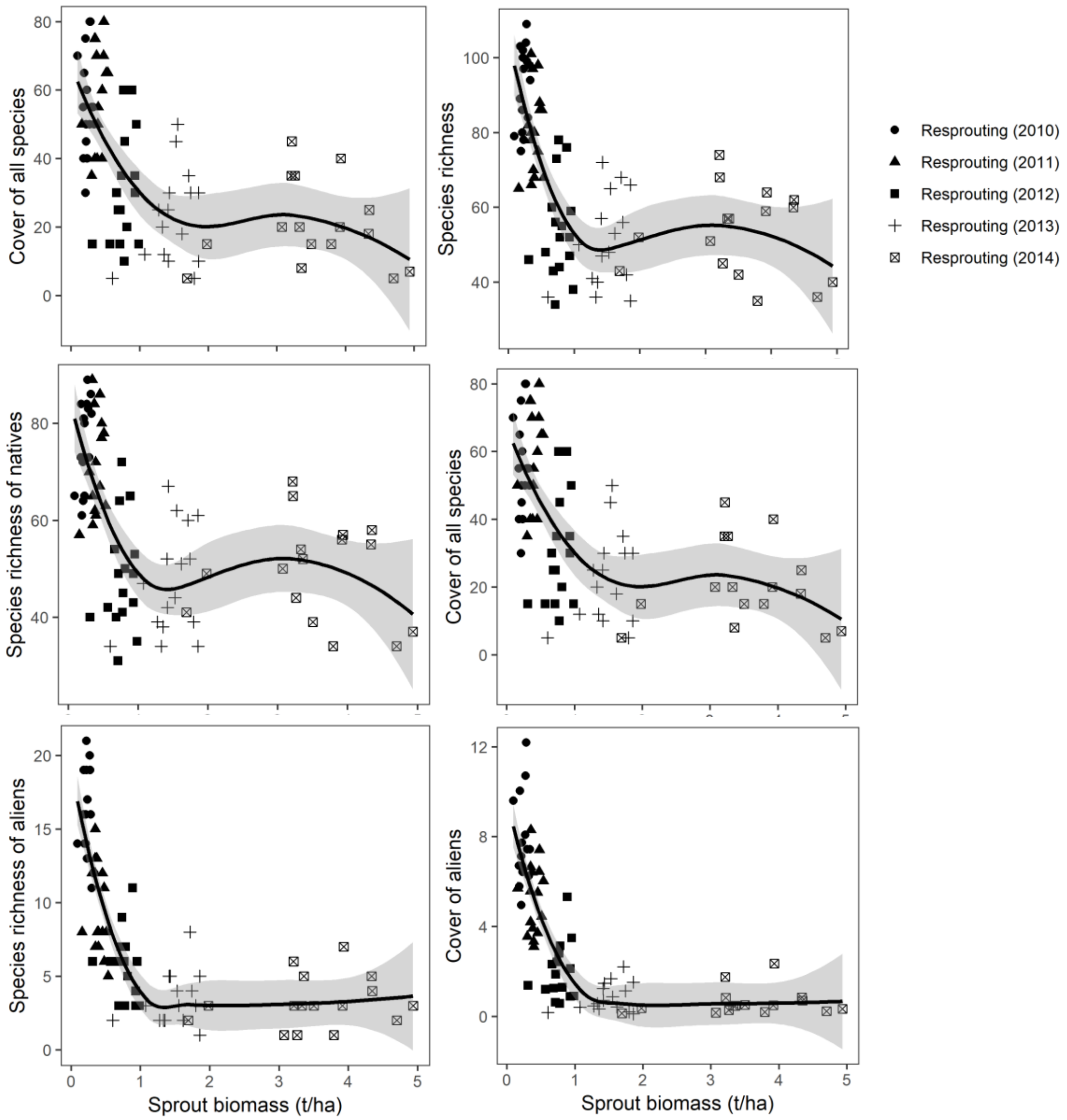

Figure S2. Relationship between sprout biomass and SR and cover of all, native and alien herbaceous species. The solid line within the grey zone represents a fitted loess model with 95% confidence intervals.

Table S2. Effects of EIVs on SR and cover of all, native and alien herbaceous plants.

Response variable	Explanatory variable (EV)	All			Natives			Alien		
		Coef.	R2	delta AIC	Coef.	R2	AIC	Coef.	R2	AIC
Species richness	Light	0.31	0.78	0.0	0.26	0.70	0.0	0.73	0.77	0.0
	Fertility	0.27	0.45	92.2	0.21	0.38	78.7	0.71	0.60	34.5
	Temperature	0.26	0.26	216.3	0.20	0.24	144.1	0.58	0.22	146.8
	Moisture	0.20	0.26	243.3	0.16	0.24	156.7	0.40	0.27	148.7
Cover	Light	0.59	0.60	0.0	0.55	0.57	0.0	0.62	0.65	0.0
	Fertility	0.40	0.25	59.8	0.35	0.21	57.0	0.63	0.55	35.9
	Temperature	0.30	0.18	67.8	0.28	0.17	62.7	0.43	0.32	75.3
	Moisture	0.28	0.13	76.3	0.25	0.12	70.7	0.43	0.26	100.6
